# TBMap: a taxonomic perspective on the phylogenetic database TreeBASE

**DOI:** 10.1186/1471-2105-8-158

**Published:** 2007-05-18

**Authors:** Roderic DM Page

**Affiliations:** 1 Division of Environmental and Evolutionary Biology, Institute of Biomedical and Life Sciences, Graham Kerr Building, University of Glasgow, Glasgow G12 8QQ, UK

## Abstract

**Background:**

TreeBASE is currently the only available large-scale database of published organismal phylogenies. Its utility is hampered by a lack of taxonomic consistency, both within the database, and with names of organisms in external genomic, specimen, and taxonomic databases. The extent to which the phylogenetic knowledge in TreeBASE becomes integrated with these other sources is limited by this lack of consistency.

**Description:**

Taxonomic names in TreeBASE were mapped onto names in the external taxonomic databases IPNI, ITIS, NCBI, and uBio, and graph *G *of these mappings was constructed. Additional edges representing taxonomic synonymies were added to *G*, then all components of *G *were extracted. These components correspond to "name clusters", and group together names in TreeBASE that are inferred to refer to the same taxon. The mapping to NCBI enables hierarchical queries to be performed, which can improve TreeBASE information retrieval by an order of magnitude.

**Conclusion:**

TBMap database provides a mapping of the bulk of the names in TreeBASE to names in external taxonomic databases, and a clustering of those mappings into sets of names that can be regarded as equivalent. This mapping enables queries and visualisations that cannot otherwise be constructed. A simple query interface to the mapping and names clusters is available at .

## Background

TreeBASE [1,2] is a database of published phylogenetic trees and associated data matrices (such as sequence alignments). It differs from other phylogenetic databases, such as PANDIT [3] and TreeFam [4], in being primarily a collection of evolutionary trees for organisms, rather than for gene families. Although it contains only a small fraction of the evolutionary trees published to date, the database is continually growing, in part because a number of journals either require or encourage authors to submit their data sets and trees to TreeBASE. In addition to supporting simple text searches to retrieve data, TreeBASE has tools for searching based on tree similarity [5] and for constructing supertrees [6].

The phylogenies stored in TreeBASE provide a wealth of information on organismal phylogeny, as well as a resource for studies on the relative merits of different sources of data [7], the shape of evolutionary trees [8,9], and methods for querying trees [5,10,11]. However, research that relies on aggregating results from different studies in TreeBASE, such as supertree construction [12], or integrating studies in TreeBASE with data elsewhere, such as information on nucleotide sequences, taxonomy, geography, and ecology is greatly hindered by the lack of an adequate taxonomic framework for TreeBASE. This is due to inconsistencies both within TreeBASE, and between TreeBASE and other databases [13,14]. Not only do these inconsistencies hinder biological investigation, they also limit the effectiveness of computational challenges, such as constructing a supertree from all green plant phylogenies in TreeBASE [15].

In this paper I describe the construction of a database, TBMap, that maps the bulk of the names in TreeBASE to names in one or more external taxonomic databases. As well as being a resource for users of TreeBASE, TBMap is intended to demonstrate the importance for phylogenetic databases of adequately handling taxonomic names – the taxonomic mapping in TBMap enables queries and visualisations that cannot otherwise be constructed using the existing version of TreeBASE.

### Why taxonomy matters

To adequately handle taxonomic names, a phylogenetic database should ensure internal and external consistency of names, be able to resolve synonymy, and be able to perform hierarchical queries [14,16].

#### Internal consistency

The first criterion of internal consistency is an obvious requirement. If multiple names are used for the same taxon, then a simple search for all data relevant to a given taxon cannot be guaranteed to have found all those data – some might be associated with an alternative name for that taxon. Examples in TreeBASE include alternative spellings of the same name (e.g., *pleistodontes greenwoodi*(sic) versus *Pleistoodntes greenwoodi*), or the same name with (*Diomedea antipodensis *AF076047) or without (*Diomedea antipodensis*) a GenBank accession number appended. These examples are issues of data quality – ideally they would be caught when the data are first entered.

However, instances of multiple names may be due to taxonomic synonymy. As our phylogenetic knowledge of a group of organisms grows it is not uncommon for this new understanding to be reflected in taxonomic changes. Consequently, names used for the same taxon in successive studies submitted to TreeBASE may have changed since the first study was submitted. For example, TreeBASE study S754 [17] uses the names *Coursetia heterantha *and *C. weberbaueri *for two species of plant that were subsequently moved to the genus *Poissonia *in TreeBASE study S813 [18], becoming *Poissonia heterantha *and *P. weberbaueri*, respectively. TreeBASE treats both sets of names as entirely distinct, failing to recognise that they are synonyms [19].

#### External consistency

The second criterion of external consistency assumes that we want to be able to apply knowledge obtained from the phylogenetic database to other domains. For example, a user wanting to employ phylogenetic methods to analyse the evolutionary ecology of a group of organisms should be able to use the same scientific name to obtain both phylogenetic and ecological data.

#### Synonomy

Achieving consistency is complicated because the same taxon may have multiple names (synonyms). As we have seen, synonymy can affect internal consistency if different studies use different names for the same taxa. It can also hamper efforts to integrate data in TreeBASE with external data, particularly if names changes occur after data has been submitted to TreeBASE. To illustrate, TreeBASE contains data for the frog *Rana pipiens *submitted as part of study S1186 in 2005 [20]. The following year Frost et al. [21] renamed this frog *Lithobates pipiens*. Users adopting Frost et al.'s classification will struggle to retrieve data about this frog, unless they are aware of its other name [22]. Ideally, phylogenetic databases would keep abreast of name changes, and be able to expand queries to include synonyms [16].

#### Hierachy

The final criterion of hierarchy is equivalent to requiring an ontology that specifies the relationships between terms. For example, as text strings, "Gallus gallus" and "Struthio camelus" have no obvious connection, but both are names of birds (class Aves). If we query a phylogenetic database using the term "Aves", we should be able to retrieve all studies containing birds, regardless of whether those studies actually contain a taxon labelled "Aves."

### Mapping TreeBASE names

In order to add a taxonomic framework to TreeBASE, I set out to map as many of the names in TreeBASE as possible to a name in an external taxonomic database. Mapping every name was not the goal, in part because not every name in TreeBASE is an organismal name. Examples include the protein fold categories drawn from the SCOP database [23] that serve as "taxa" in study S909 [24].

#### Previous work

NCBI's LinkOut [25] feature provides a basic mapping of its names to TreeBASE, based primarily on exact string matches. However, less than half the names in TreeBASE have an exact match in NCBI (see below). Furthermore, string matching by itself is not enough because of the existence of homonyms – the same name can be used in different nomenclatural codes [26]. Naive string matching can lead to animal taxa in NCBI being erroneously linked to plants in TreeBASE (and visa versa) [27].

Herbert et al. [28] have used the BIO-AJAX tool to clean taxonomic names in TreeBASE, using the NCBI Taxonomy [29]. The mapping work described here differs from Herbert et al. in several respects – I use more than one taxonomic database, make use of additional information in the names (such as GenBank accession numbers), use approximate string matching, and also a degree of manual inspection to detect homonyms.

## Construction and content

number appended). The components of this graph are "name clusters."

**Figure 1:**
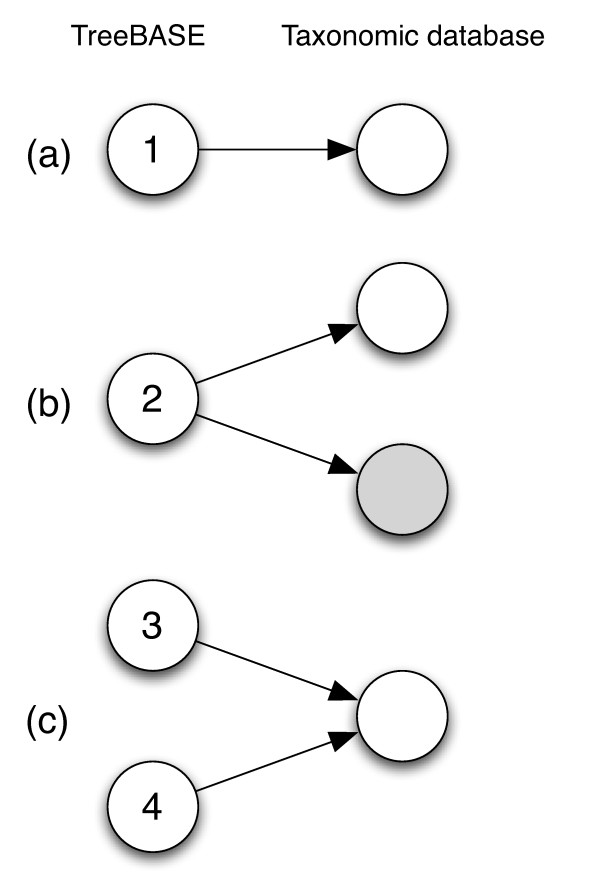
**Matching names in the TaxonName field in TreeBASE to one or more taxonomic name databases**. In (*a*) TaxonName matches a name in an external database; in (*b*) TaxonName occurs in two different databases. (*c*) shows a case where two different TaxonNames match the same name in a taxonomic database (for example, TaxonName 3 may be a taxon name, and TaxonName 4 is the same name but with a GenBank accession number appended). The components of this graph are "name clusters."

We can model the problem of matching TreeBASE names to taxonomic names in external databases using a bipartite graph, *G*, where the nodes are partitioned into two disjoint sets, one representing all the names in TreeBASE, the other representing names in the taxonomic databases (Fig. 1) that have been matched to one or more names in TreeBASE. The edges of the graph (*u*, *v*) represent the mapping of a name in TreeBASE onto a name in an external database. These edges are labelled by a description of the kind of match, for example whether the TreeBASE name is an exact or an approximate match. Once the mapping has been made, the components of the resulting graph correspond to "name clusters", i.e., sets of TreeBASE taxon names that are equivalent. For example, in Fig. 1 the four TreeBASE names belong in three components: {1}, {2}, and {3, 4}, hence TreeBASE names 3 and 4 are part of the same name cluster, and hence refer to the same taxon.

### Construction

To construct *G*, a list of 52778 names was obtained from TreeBASE using a CGI script. For each name TreeBASE stores the primary key TaxonID and a text string TaxonName. Both are unique within TreeBASE. Using a variety of methods listed below, names stored in the TaxonName field of TreeBASE were matched to names in external databases. For each "hit" I recorded the name of the source database, the unique identifier in that database, the name as stored in that database, and the nature of the match (e.g., whether exact or approximate).

#### Exact matching

The original TreeBASE names were searched for in taxonomic databases [29-32]. In the case of NCBI Taxonomy [29] and ITIS [30], the databases were downloaded and imported into a MySQL database where a simple table join was done to find exact matches. For databases that could not be downloaded, the web interface or services provided by that database were used directly, or via the Taxonomic Search Engine [27]. Exact matches were flagged as "exact".

#### Cleaning names

Numerous names in TreeBASE result from concatenating the taxon name and a string which may or may not be an additional identifier. Examples include GenBank accession numbers, such as "*Moringa drouhardii *AF378628", voucher specimen codes, such as "*Eleutherodactylus *sp. B FMNH257689 Panama"), or symbols representing a sample number. These will prevent exact string matching from finding the corresponding name in a taxonomic database. I used various methods to cope with this, such as "cleaning" the names to extract taxonomic names, or extracting the additional information and trying to interpret it.

#### uBio findIT web service

Names were cleaned by using uBio's findIT SOAP service, which uses algorithms derived from TaxonGrab [33] to extract taxonomic names from text. For each taxon name findIT returns a canonical version of the name and, if the name is present in uBio, a namebankID for that name. These canonical names were used in subsequent database searches. These searches were restricted to cases where the canonical name differed from the TreeBASE names, and these matches were flagged as "substring".

#### Substring matching

The findIT algorithm is designed to extract taxonomic names from a broad range of sources, and initial experiments suggested it could make errors, often by removing too much of the name string. To complement this algorithm, all TreeBASE names were matched against a simple regular expression

/\w+\s(cf.\s)?([a-z]+(-[a-z]+)?)+((\s(var|ssp).?)?\s[a-z][a-z]+)?/

that removed extraneous numbers and codes, such as "cf.", "var.", "ssp.". The resulting substrings were cleaned of any other obviously extraneous strings (e.g., "clone", "ex", "from", "on") and matched against the source databases, and hits were flagged as "substring".

#### GenBank accession numbers

Names that might contain accession numbers were identified by testing whether the TaxonName matched the regular expression /(A[A-Z][0-9]?[0-9]{5}|[A-Z][0-9]?[0-9]{4})/, and the NCBI Taxonomy ID for the corresponding GenBank accession number was retrieved from NCBI. These hits were flagged as "accession". This regular expression matches strings that start with one or two letters (A followed by any other letter) followed by 5 or 6 digits, and hence matches GenBank accession numbers such as X12841 and AF308702. GenBank has recently started using an expanded set of letters in the prefix for accession numbers, so this expression will not match these. Furthermore, not all strings matching this regular expression are accession numbers, either through typographic errors (e.g., in "*Mycosphaerella africana *AF28369", TaxonID T16433, there is a '0' missing from the accession number [GenBank: AF283690]), or because a specimen or voucher code appended to the name coincidentally resembled an accession number.

#### Museum specimen codes

Specimens housed in museum collections typically include standardised museum abbreviations, and these can be readily identified. For example, FMNH 257689 identifies a specimen from the Field Museum of Natural History, Chicago. Specimen information is sometimes recorded in the paper publishing the study, and/or the GenBank record for the sequence in TreeBASE, enabling the TreeBASE name to be linked to the corresponding NCBI Taxonomy name. These hits were flagged as "substring".

#### Approximate string matching

In addition to extraneous symbols, names can be misspelt, or have alternative spellings. To accommodate this, names that had been cleaned using the substring regular expression, but which were not found in the taxonomic databases were processed using the agrep approximate string matching tool [34]. Each name was matched against names from NCBI, allowing a maximum of two mismatches. Initial experiments suggested that matching uninomials (taxonomic names comprising a single word) generated a high number of spurious matches, so only names with two or more parts were processed. Hits found using agrep were flagged as "agrep".

#### Manual mapping

Where feasible, cases where automated mapping produced no results were investigated "by hand", involving searches of Google, and consulting the literature. The results of the automatic mapping were also reviewed. Given the scale of the task (several thousand names remained unmatched), this work is ongoing. Approximate matches found by inspection were flagged "approx". A handful of difficult cases which were resolved only after consulting the literature were flagged "manual".

#### Synonymy

nodes corresponding to TreeBASE taxon names are labelled with the corresponding TaxonName and TaxonID. Names in external databases are represented by the database name and the unique identifier used within that database, e.g. "ncbi:168522" is tax_id 168522 in the NCBI Taxonomy database.

Given that the same taxon may have more than one name, it may be that two name clusters in the graph *G *are, in fact, the same taxon. To accommodate this, we can add to the graph *G *edges between nodes representing names that are synonyms. A complication is that there are different notions of synonymy, and different databases model synonymy in different ways. NCBI uses a single unique identifier for all the names that apply to a taxon, whether that name is valid, a synonym, or a vernacular ("common") name. For example, the sperm whale is known as both *Physeter catodon *and *Physeter macrocephalus*. Both names in NCBI have the tax_id 9755. Other databases may have distinct identifiers for each name (such as tsn:180489 and tsn:180488, respectively in ITIS).

We can divide synonyms into "homotypic" and "heterotypic". Homotypic synonyms are names that share the same type (either type specimen for a species-level name, or a type taxon for a genus or family name), and hence objectively refer to the same taxon. Heterotypic synonyms are based on different types, and hence whether they refer to the same taxon is a matter of inference or taxonomic opinion. Although homotypic and heterotypic synonyms are also referred to as "objective" and "subjective'' synonyms, the distinction between objective and subjective is not absolute, in the sense that there can be uncertainty about whether two authors were actually referring to the same type specimen, and we can establish beyond reasonable doubt that two names with different types are the same taxon (for example, sequence identity in different life history stages of the same organism, such as the anomorph and telomorph stages in fungi). Nomenclatural databases such as IPNI provide objective synonyms. Databases such as ITIS and NCBI provide a mixture, without clearly distinguishing between the two. In order to minimise subjective synonyms (for example, whether two family-level taxa should be merged into a single family), I did not include NCBI synonyms "in-part" or "includes" in the mapping, and ITIS synonyms were only added if they were at the level of species or below.

**Figure 2:**
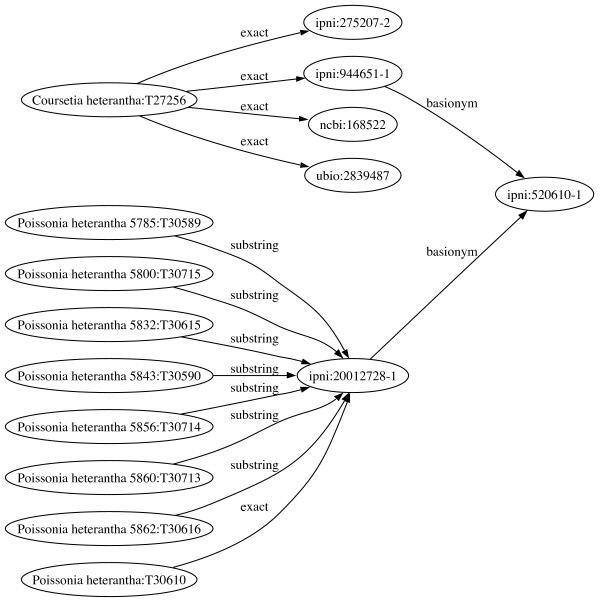
**Adding information on synonymy links name clusters**. TreeBASE contains a taxon called *Coursetia heterantha*, and eight variations on the name *Poissonia heterantha*. Based on mapping strings these names form two distinct clusters. However, the two names share a common basionym, *Tetraphrosa heterantha *(IPNI record 520610-1), and hence are synonyms. Adding this information to the graph links these two otherwise disjoint name clusters. In the graph, nodes corresponding to TreeBASE taxon names are labelled with the corresponding TaxonName and TaxonID. Names in external databases are represented by the database name and the unique identifier used within that database, e.g. "ncbi:168522" is tax_id 168522 in the NCBI Taxonomy database.

Additional synonymy information for plant names was obtained from the IPNI web site [31]. A query URL was generated for each IPNI identifier in the mapping, and the resulting HTML was scraped to extract links to other names. For each synonym, an edge was added to the graph *G *linking the two IPNI names, and the edge was labelled with the type of synonymy, i.e., "basionym", "basionym of", "nomenclatural synonym", "replaced synonym", or "replacement synonym". IPNI does not always explicitly state synonymies between names, but usually provides enough information for this relationship to be inferred [19]. For example, the IPNI database contains the names *Coursetia heterantha *and *Poissonia heterantha*, and gives *Tetraphrosa heterantha *as the basionym (original name) for both names. Hence, *Coursetia heterantha *and *Poissonia heterantha *are synonyms (Fig. 2).

#### Name clusters

Name clusters were extracted from *G *by finding all components of *G*. Each component was given an identifier by appending "TC" and the number of the smallest TaxonID of a TreeBASE name in the cluster. The corresponding TaxonName serves as the label. For example, the cluster shown in Fig. 2 has the identifier "TC27256", and the label "Coursetia heterantha".

### Mappings

The database comprises 1,071,133 mappings between 45,509 TreeBASE TaxonName fields and 89,947 names from extant databases. Of the 52,778 names in the version of TreeBASE used, 7269 names remain unmapped. Table 1 summarises the mappings. NCBI supplied the single largest source of names, followed by uBio. The total for IPNI is inflated relative to the other databases because it combines three databases, hence a single name may be mapped to up to three distinct IPNI identifiers. The bulk of the mappings were exact matches, but a large fraction of TreeBASE names were mapped using substring or approximate matching. The approximate string maps show that some 6% of names in TreeBASE are misspelt.

**Table 1:** Mappings between TreeBASE and taxonomic databases

Mappings between TreeBASE and taxonomic databases							
Source	Exact	Substring	Approximate	Accession	Manual	Synonym	Total

IPNI	22271	3084	28		6		25389
ITIS	11314	511			6		11831
NCBI	24273	11580	2914	1850	35	933	41585
uBio	28038	257	21		4		28320
other	3				5		8

Totals	85899	15432	2963	1850	56	933	107133

For each database the table lists the category of mapping. The category "synonym" refer to names that NCBI labels as "anamorph", "equivalent", "in-part", "includes", and "synonym." The source "other" comprises names that were not found in any of the four taxonomic databases.

### Clusters

The graph *G *contained 32817 clusters, the largest of which comprised 416 TaxonIDs. The clustering reduces the number of taxa in TreeBASE by 12,692 (27.9%), which is a measure of the impact of cleaning names on TreeBASE.

### Hierarchical queries

**Figure 3:**
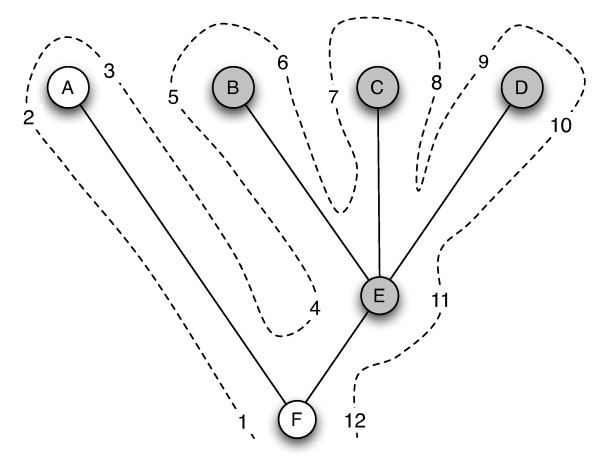
**Nested sets representation of a tree**. To generate the nested set representation of a tree the tree is traversed in depth-first order (dotted line), and each node is assigned a pair of numbers that record the order in which that node is visited. The left number records the first time the node is visited, the right number records the last visit. The set of nodes in a given subtree correspond to those nodes whose left and right visitation numbers fall within the range for the root of the subtree.

To support hierarchical queries the NCBI taxonomy tree was imported into the MySQL database, and converted into a "nested sets" representation [35] using a custom C++ program. This representation assigns a pair of numbers to each node in the tree that records the order in which the node is visited during a depth-first traversal of the tree (Fig. 3). The subtree rooted at a node *n *can be recovered by finding all nodes whose visitation numbers lie within the range of the left and right visitation numbers of node *n*. For example, in Fig. 3 the subtree rooted at node "E" corresponds to all the nodes in the tree with left visitation number ≥ 4 and right visitation number ≤ 11.

## Utility

The mapping between TreeBASE and external name sources enables us to query TreeBASE in new ways, and facilitates new visualisations.

### Impact on retrieval

To evaluate the impact on retrieval of adding a classification to TreeBASE, I examined a query log for TreeBASE supplied by Bill Piel. The log comprises 358314 queries made over the period December 1998 to March 2006. There were 63,398 distinct query terms, submitted from 60,063 distinct IP addresses. From this log the top 30 query terms were obtained, and two searches performed. The first is a simple SQL text search of a local copy of TreeBASE, finding all studies that contain a taxon matching the query term. This approximates the current taxonomic name search in the online version of TreeBASE. The second search looked for the corresponding name in the NCBI classification and if found, used the nested set representation of the NCBI classification to find all studies containing taxa in the subtree rooted at that node.

The results are shown in Table 2. In all cases the hierarchical search found more studies than the simple search. For example, although only five studies in TreeBASE contain the taxon name "Aves" (birds), TreeBASE contains an additional 23 studies on birds. For higher taxonomic groups such as fungi and angiosperms, the improvement is even more substantial.

**Table 2:** Effectiveness of string and hierarchical queries

TreeBASE studies retrieved by text and hierarchical queries					
Rank	Term	Frequency	tax_id	Text	Hierarchical

1	homo sapiens	9700	9606	22	25
2	mammalia	5028	40674	7	69
3	Fungi	1875	4751	6	440
4	**angiosperms**	1825	3398	8	470
5	pine	1772			
6	carnivora	1723	33554	6	18
7	maple	1666			
8	acer	1618	4022	5	7
9	chordata	1373	7711	1	140
10	Agaricus bisporus	1286	5341	17	18
11	Homo	1212	9605	1	26
12	oak	1103			
13	Cetacea	1006	9721	9	15
14	bacteria	988	2	2	21
15	pinus	973	3337	4	14
16	Candida albicans@	963	5476	21	21
17	**human**	951	9606		25
18	Zea mays	929	4577	13	17
19	donoghue	914		-	-
20	jody hey	901		-	-
21	Aves	888	8782	5	28
22	quercus	865	3511	1	3
23	chase	804		-	-
24	Hibbett	803		-	-
25	drosophila	792	7215	3	15
26	Drosophila melanogaster	727	7227	13	13
27	Nematoda@	649	6231	3	29
28	arthropoda	648	6656	5	148
29	primates	619	9443	2	28
30	mollusca	610	6447	4	38

Total		45211		158	1628

For the 30 most common query terms entered by users of TreeBASE between December 1998 to March 2006, the table shows the frequency of that term, the NCBI taxonomy tax_id (where applicable), and the number of studies retrieved by a SQL text query and by a hierarchical query using a nested sets representation of the NCBI classification (Fig. 3). Queries in **boldface **are vernacular names that are also present in the NCBI taxonomy database. Two queries used the '@' symbol, which TreeBASE treats as a wild-card. For those terms the corresponding SQL text query used the '%' wild-card.

### Visualisation

**Figure 4:**
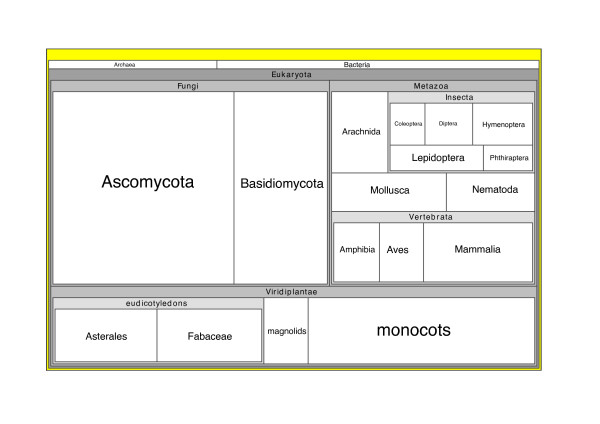
**Treemap of TreeBASE**. Overview of the relative abundance of different taxonomic groups in TreeBASE. Each panel in the treemap represents a taxonomic group in the NCBI classification, scaled proportionally to the number of studies containing that taxon in TreeBASE. Diagram generated using the program Treemap 4.1 [54].

The problem of visualising large hierarchies has spawned a large literature [36-38] Given a mapping onto a classification, we can now explore the utility of these visualisations in the context of TreeBASE. Fig. 4 shows a treemap for studies in TreeBASE, which could form the basis of an alternative graphical interfaces to TreeBASE – the user to click on a panel and go to studies containing the corresponding taxon.

Treemaps can also be used to categorise results from searches [39], so that the user immediately sees the taxonomic distribution of query results. They also have potential to identify taxa that are under-represented in TreeBASE. At a glance we can see that TreeBASE studies are primarily of eukaryotes, approximately equally split between fungi, plants and animals. Given that the bulk of the described taxa are animals (particularly arthropods), it is clear that taxa such as insects are grossly under-represented in this database.

## Discussion

### Problematic names

Some names in TreeBASE proved problematic to map, for reasons such as errors in the name or in external taxonomic databases, homonyms (different taxa with the same name), alternative spellings of the same name, undescribed taxa, and complicated synonomies.

#### Accession numbers

Some accession numbers in TreeBASE taxon names are incorrect, such as T16449, which is listed as "*Botryosphaeria dothidea *AF283577". Sequence [GenBank:AF283577] is actually a snake cytochrome *b *sequence (from *Pantherophis obsoletus*). The accession number is presumably [GenBank:AF283677], which is from *Botryosphaeria dothidea*.

#### Homonyms

Homonyms can cause problems both within and between databases. Within TreeBASE, two taxa have the name "Proboscidea", one of which (T7290) is the mammalian order Proboscidea (elephants), and the other (T3002) is plant genus *Proboscidea*. TreeBASE itself confuses the two taxa – Study S1222 on mammal phylogeny includes the order Proboscidea in matrix M2121, but uses the TaxonID T3002 corresponding to the plant genus *Proboscidea*. Another example is *Daubentonia*, which is a legume (plant) genus and a lemur (animal) genus. TreeBASE contains a single TaxonID for this name (T954), which means that studies S11x6x95c08c52c19 [40] and S1060 [41] mistakenly share the same taxon.

#### Alternative spellings

There is not always unanimity regarding the correct spelling of a taxonomic name, hence mismatches between TreeBASE and external databases may reflect genuine disagreement over spelling, rather than mistakes. As an example, there is a plant genus in the family Asteraceae with two alternative spellings, *Vierea *and *Vieraea*. In TreeBASE study S1x28x96c16c45c this genus is listed as *Vierea*, whereas the NCBI Taxonomy database lists this as *Vieraea *(note the 'a' before 'ea'). IPNI [31] uses the same spelling (IPNI record 11475-1) as NCBI. However Anderburg [42], the author of study S1x28x96c16c45c lists this genus as (p. 102):

"*Vierea *WEBB & BERTH. WEBB & BERTHELOT, Hist. Nat. Isles Canar. 3 (2.2):84 (1839).-Type: *V. laevigata *WEBB. Synonym: *Vieraea *SCH.-BIP in WEBB & BERTH., corr. superfl."

indicating that he regards the spelling of *Vieraea *to be an unnecessary correction of the original spelling *Vierea*.

#### Approximate matching

Identifying the correct mapping using approximate string matching assumes that we have an authoritative list of names to match against, and that there are few names that are sufficiently similar to each other to generate false matches. Here I used the NCBI taxonomy because it is readily available, and given that most studies in TreeBASE use sequence data, it is likely that many TreeBASE names should match a name in the NCBI list. However, the NCBI taxonomy is not error free. For example, T38508 (*Apomys gracilirostris*) is not found in NCBI, but is near match to (*Apomys gracilostris*). In this case, the spelling in TreeBASE is correct, as can be verified by consulting the original publication of the name [43].

#### Undescribed taxa

Given that in some groups of organisms phylogenetic research is outpacing taxonomic description, it is not uncommon to find taxa given the epithet "sp.", for example, taxon T8341 in TreeBASE is "*Drosophila *sp.". In poorly known groups there will be many such taxa, consequently it may not be clear which undescribed species is being referred to. NCBI deals with multiple undescribed species in the same genus by appending sufficient text to "sp." to make the identifier unique. Hence, there is a "*Drosophila *sp." in NCBI (tax_id 7242), but it is not the "*Drosophila *sp." in TreeBASE. The later is stored in NCBI as "*Drosophila *sp. 'white tip scutellum' " (tax_id 58313). However, this can only be discovered by reading DeSalle and Baker [44] – who deposited the study (S320) containing taxon T8341 in TreeBASE – getting the accession number for the sequence for "*Drosophila *sp." and looking up that accession number in GenBank.

Ideally, all taxa referred to as "sp." would eventually be properly named. Hawaiian *Drosophilia *are relatively well known taxonomically, and taxon "*Drosophila *sp. 'white tip scutellum' " has subsequently been identified as *Drosophila longiperda *Kambysellis [45]. Many taxa are not so fortunate. Furthermore, *Drosophila longiperda *is itself now in NCBI under the separate tax_id 251450, so that tax_id 58313 and 251450 refer to the same taxon.

#### Synonymy

While most taxonomic name databases store information on synonyms, some difficult cases were not discovered by the combination of automated matching of names and extracting synonyms. The plant genus *Gastrolobium *provides some instructive examples, particularly T32944 *Gastrolobium ebracteolatum*. This name does not occur in NCBI taxonomy, despite TreeBASE study S875 [46] being based on nucleotide sequences. In their paper, Crisp and Chandler [46] list Genbank accession numbers [Genbank: AY015102] and [GenBank:AY015219] as coming from *Gastrolobium ebracteolatum*, whereas Genbank lists these sequences as being from *Oxylobium lineare*. Despite the fact that these two names are completely different, they refer to the same taxon. Chandler et al.'s 2001 study [47] suggested *Oxylobium lineare *be incorporated into the genus *Gastrolobium*. Normally this would result in only the generic name changing, resulting in the new combination *Gastrolobium lineare*, which at least shares some similarity with the original name. However, the name *Gastrolobium lineare *has already been taken for a different taxon, hence when Chandler et al. [48] formally moved *O. lineare *to *Gastrolobium *a new species epithet was required, yielding *Gastrolobium ebracteolatum*. There are mercifully few cases in TreeBASE as complicated as this.

### Interpreting name clusters

Scientific names are ambiguous identifiers [49] as it is not always clear that two researchers using the same name are referring to the same taxon. Name clusters can be thought of as loosely equivalent to "taxonomic concepts" [49,50], that is, a set of names and references to those names that we can regard as referring to the same biological entity. For example, the variations of *Poissonia heterantha *shown in Fig. 2 are samples of different populations of this species [18], and *Coursetia heterantha *is part of this cluster, based on the objective synonymy of the two names. The membership of *C. heterantha *is further supported by sequence [GenBank:AF398842], which is listed as being from *C. heterantha *in TreeBASE study S754 [17], then from *P. heterantha *in study S813 [18].

### Alternatives to hierarchical classifications

Hierarchical classifications are powerful tools for navigating and querying biological databases, but have their own problems. There are numerous classifications a biologist can choose from, and not all may retrieve the same set of results. If a database imposes a single classification upon its users, then that may restrict the kinds of queries that can be asked. NCBI is a good example of this – the classification of animals in the NCBI taxonomy does not reflect results of recent molecular phylogenetics [51]. Furthermore, no fully comprehensive classification of all organisms exists. The NCBI taxonomy classification used here contains very few extinct organisms, limiting its utility in navigating a database that contains fossil taxa (especially if a study contains no extant taxa).

In its current incarnation, TreeBASE tries to obviate these problems using the notion of "tree surfing", where the user can "surf" to neighbouring trees that share at least one taxon in common with the starting tree. If we model TreeBASE as a graph *G *where the nodes represent the set of taxa in a tree, and two nodes are connected by an edge if and only if the taxon sets corresponding to those nodes have *k *taxa in common, then for tree surfing to be successful at a minimum the graph *G *for *k *= 1 must be connected. It is not [52]. The mapping exercise undertaken here improves the situation somewhat, but *G *remains unconnected. Hence, studies relevant to a user's query may occur in different components of the graph, and hence will be difficult, if not impossible to discover by tree surfing. However, the addition of larger phylogenetic trees to TreeBASE is likely to improve this situation.

## Conclusion

Matching taxonomic names is more than a simple matter of string matching, it requires identifying alternative names for the same taxon (both lexical variants and synonyms), and distinguishing among uses of the same name for different taxa [16]. Mappings based on names may also be erroneous if two studies used the same name for a taxon, but differed in how they interpret that taxon. This is a general problem [49] for taxonomic databases. The problem is ameliorated somewhat in TreeBASE, given that different studies often reuse the same data (e.g., the same nucleotide sequences), which can reduce ambiguity in what the authors of those studies meant when they used a given taxonomic name.

Much of the difficulty experienced in making sense of the names in TreeBASE results from the lack of validating names when the data are input. As a consequence, much tedious effort has to be expended on *post hoc *mapping of names. An obvious improvement to TreeBASE (and any other phylogenetic database) would be to validate names when data is first input using, for example, web services that are now available [27].

In addition to a lack of input validation, the other reason the mapping is not always straightforward is that many taxon names in TreeBASE are best though of as Operational Taxonomy Units (OTUs) rather than taxonomic names. They identify a set of observations for a particular specimen, set of specimens, or a taxon. For instance, "Eleutherodactylus crassidigitus FMNH257676 Panama" (TaxonID T51971) refers to a 1200 base pair stretch of mitochondrial DNA ([GenBank:AY273113]) obtained from Field Museum Natural History specimen FMNH 257676, which has been identified as *Eleutherodactylus crassidigitus *[53]. Hence, part of the problems faced by the current version of TreeBASE could be ascribed to inadequately modelling the relationship between the entities it stores – it does not cleanly separate names from OTUs. Although a necessarily tedious undertaking, the benefits of having an explicit mapping between TreeBASE names and external databases is reflected in the improved retrieval of studies when querying using higher-level taxonomic names. The most significant improvements are in large taxonomic groups, such as angiosperms and fungi which make up the bulk of the database (Fig. 4), where hierarchical queries retrieve two orders of magnitude more studies (Table 2) than simple text queries.

## Availability and requirements

The database can be freely accessed at  using any modern web browser.

## Abbreviations

• FMNH, Field Museum of Natural History

• IPNI, International Plant Names Index

• ITIS, Integrated Taxonomic Information System

• NCBI, National Center for Biotechnology Information

• uBio, Universal Biological Indexer and Organiser
